# Characterization of Newcastle Disease Virus and poultry-handling practices in live poultry markets, Ethiopia

**DOI:** 10.1186/2193-1801-3-459

**Published:** 2014-08-23

**Authors:** Delesa Damena Mulisa, Menbere Kidane W/Kiros, Redeat Belaineh Alemu, Melaku Sombo Keno, Alice Furaso, Alireza Heidari, Tesfaye Rufael Chibsa, Hassen Chaka Chunde

**Affiliations:** National Animal Health Diagnostic and Investigation Center, Sebeta, Ethiopia; Research and Innovation Department, Istituto Zooprofilattico Sperimentale delle Venezie, OIE/FAO and National Reference Laboratory for Newcastle Disease and Avian Influenza, OIE collaborating Center for Diseases at the Human-Animal Interface, Legnaro, Padova, Italy

**Keywords:** Newcastle disease virus, Biosecurity practices, Molecular characterization, Live poultry markets, Ethiopia

## Abstract

Newcastle disease represents the most severe poultry disease responsible for marked economic losses in Ethiopia. To provide a molecular characterization of Newcastle disease viruses circulating in this country, a cross sectional survey was conducted at five selected live poultry market sites in Addis Ababa. In addition, baseline data on the live poultry market system were acquired through a detailed questionnaire submitted to poultry traders. We identified 44/146 positive samples, 29 of which were virulent strains belonging to sub-genotype VIf. The very poor biosecurity practices, which have resulted from responses of the participants, suggest that they might have had a heavy impact in the spread of the disease. This study provides important information on epidemiology and control of NDV in Ethiopia and highlights the importance of implementing surveillances and biosecurity practices in live poultry markets.

**Electronic supplementary material:**

The online version of this article (doi:10.1186/2193-1801-3-459) contains supplementary material, which is available to authorized users.

## Introduction

Poultry play an important economic, nutritional and socio-cultural role in the livelihoods of poor rural households in developing countries, including Ethiopia. The total poultry population in Ethiopia is estimated at 43 million, 97% of which are village chickens (Central Statistical Agency 2012). Poultry rearing is particularly important to women, who often own and manage chickens and the resulting income is often used to support education of children. Despite its role in raising incomes and reducing poverty in local communities of Ethiopia, poultry production is hampered by wide arrays of constraints among which infectious diseases, such as: Newcastle disease, Infectious Bursal Disease, Mycoplasmosis, Pasteurellosis and Salmonellosis, are the major ones (Chaka et al. 2012).

Newcastle disease(ND) is one of the major problems in village chickens in most parts of Ethiopia (Nasser 1998; Tadelle and Jobre 2004; Mazengia 2012). The disease has become endemic in poultry population and recurs every year inflicting heavy losses (Tadelle and Jobre 2004). The highest rate of ND outbreaks from March to May is suggested to be associated with high rate of chicken marketing for Easter (Spradbrow 1999). The main movement of chicken marketing is from periphery to the center (rural to towns) which favors the spread of diseases all over the country (Dessei and Ogle 2001). However, there is acute lack of information on bio-security situation and roles of poultry marketing practices in diseases dissemination in the country (Shewantasew et al. 2012).

ND is caused by virulent strains of Newcastle diseases virus (NDV) or avian paramyxovirus type 1 (APMV-1) that belongs to the genus *Avula* and family *Paramyxoviridae* (Mayo 2002). NDV has an enveloped, single-stranded negative sense RNA genome of approximately 15 kb that contains six genes encoding major structural proteins such as: nucleocapsid protein (NP), phosphoprotein(P), matrix protein(M), fusion protein(F), hemagglutinin-neuraminidase(HN), and the RNA- dependent RNA polymerase(L) (Kattenbelt et al. 2006) as well as two additional proteins, V and W from the P gene by a mechanism called RNA editing (Steward et al. 1993).

The key contributors to NDV pathogenicity are the formation of an active fusion protein upon cleavage of the F protein precursor (F0) as well as the presence of a number of basic residues in the fusion protein cleavage site (Toyoda et al. 1987; Glickman et al. 1988).

NDV that are virulent for chickens have a multibasic amino acid sequence ^112^R/K-R-Q-K/R-R^116^ at the C–terminus of the F2 protein and F (phenylalanine) at residue 117, which is the N-terminus of the F1 protein, whereas the viruses of low virulence have a monobasic amino acid sequences in the same region of ^112^G/E-K/R-Q-G/E-R^116^ and L (leucine) at residue 117 (Kim et al. 2008; Office of International Epizootes 2012).

Although ND represents the most severe poultry disease responsible for marked economic losses in Ethiopia, virological and epidemiological information concerning the virus strains circulating in the country especially in village chickens are extremely scarce. The aim of this study was therefore, to detect NDV and to perform a molecular characterization of the virulent strains currently circulating at five live poultry market sites in Addis Ababa. In addition, we investigated the biosecurity practices implemented at the market sites to identify the factors that can contribute to the spread of the virus.

## Materials and methods

### Sample origin

**Figure 1:**
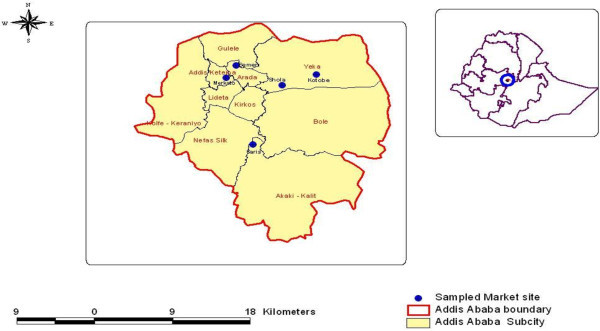
**Map of Study area.** Poultry market sites where samples were collected.

The study was conducted in December 2012 in five live poultry market sites in Addis Ababa namely: Kotebe, Merkato, Shola, Saris and Semen (Adisu gebeya) (Figure 1). These are among the biggest poultry market sites in Addis Ababa that host chickens originating from most parts of the country, mainly from places located within 300 kms radius such as: Sodo, Hosaina, Dessie, Shashemane, Jimma, and Ambo. All the markets are outdoor markets where chickens are kept in wire mesh cages, each housing up to 40 birds during the day and night.

### Sampling, data collection and virus isolation

A total of 365 chickens were sampled: From each market, 70 birds were sampled except Merkato from where 85 birds were sampled by considering its larger size compared to the others. Thus, 73 tracheal swabs (pool of five) and 73 cloacal (pool of five) were collected. In separate cryovial containing 2 ml of freshly prepared viral transport media (VTM).

The swabs were collected in pairs (Tracheal swab pools and Cloacal swab pools were always collected from the same chickens). After sampling, the specimens were transported to the National Animal Health Diagnostic and Investigation Center (NAHDIC) laboratory and stored at -80°C until processing. Besides, a standardized questionnaire was administered to the poultry traders in each market sites. A total of 100 volunteer traders were interviewed to assess the bio-security practices in live poultry markets. The questionnaire included questions about the source of the birds, means of transportation, the number of birds in each market site, cleaning practices at the market sites including the cages, health status of the birds and disposal of manures and dead chickens.

Viruses were isolated from the swab samples by standard virus isolation methods in embryonated chicken eggs. Three embryonated chicken eggs of 9 to 11-day-old per sample were used for inoculation (Alexander and Senne 2008). The inoculated eggs were candled every 24 hours to check embryo vitality. Eggs containing dead embryo on each day and those remained at the end of incubation period, were removed from incubator and chilled at +4°C overnight. Allantoic fluids were harvested and tested by Haemagglutination (HA) test for its ability to haemagglutinate chicken RBCs.

### Haemagglutination and Haemagglutination Inhibition tests

The HA assay was carried out in microtitre plate as outlined by the World Organization for Animal Health (Office of International Epizootes 2012). HA positive samples were tested by Heamaglutination Inhibition (HI) reaction to specifically determine the haemagglutinating agents. Inactivated antigen for HA test and positive and negative sera for HI controls were obtained from Istituto Zooprofilattico Spermentale delle Venezie (OIE/FAO Reference Laboratory for AI and ND), Padua, Italy.

### Polymerase Chain Reaction tests

Forty four HA and HI positive allantoic fluids were analyzed by real- time reverse transcription (RT-PCR) tests. Viral RNA extraction from HI positive allantoic fluid was conducted using Qiagen® RNeasy Mini kit according to manufacturer’s instruction. Real-time RT-PCR reactions were performed at National Animal Health Diagnostic Center, Sebeta using an Applied Biosystems 7500 Fast Real-Time PCR thermo cycler. A primer probe combination from conserved region of M gene APMV-1 F M + 4100 5’-AGT GAT GTG CTC GGA CCT TC-3’, APMV-1 R M-4220 5’-CCT GAG GAG AGG CAT TTG CTA-3’ and Probe APMV-1 Probe M + 4169 5’- FAM- TTC TCT AGC AGT GGG ACA GCC TGC BHQ-3’ (Wise et al. 2004) was used to amplify and detect all NDV isolates. The M-gene positive samples were re-run by a second primer-probe set targeting the F gene Forward F + 4829 5’-GGT GAG TCT ATC CGG ARG ATA CAA G-3’, Reverse Primer F- 4939 5’-AGC TGT TGC AAC CCC AAG-3’ and Probe F + 4894 5’-FAM-AAG CGT TTC TGT CTC CTT CCT CCA-BHQ-3’ (Wise et al. 2004) to specifically amplify and detect only pathogenic strains of NDV. Twenty nine F gene Positive allantoic fluids were submitted to Instito Zooprofilattico Sperimentale delle Venezie (OIE/FAO Reference Laboratory for AI and ND), Padua,Italy for further analyses.

### Nucleotide Sequencing and phylogenetic analysis

Total RNA was extracted from infective allantoic fluids using Qiagen® RNeasy Mini kit according to manufacturer’s instruction. Amplification was performed with primers NOH-For (5’-TAC ACC TCA TCC CAG ACA GG-3’) and NOH- Rev (5’-AGT CGG AGG ATG TGT TGG CAG C-3’) which encompass 260 bp region of the fusion (F) gene. Amplicons were then purified with ExoSAP-IT (USB Corporation, Cleveland, OH). Sequencing was carried out by using Big Dye Terminator v 3.1 cycle sequencing kit (Applied Biosystems, Foster City, CA, USA) in a 16-cappilary ABI PRISM 3131x Genetic Analyzer (Applied Biosystems, Forster City, CA,USA).

For complete sequencing of F gene, RNA was reverse transcribed with the Superscript III Reverse Transcriptase kit (Invitrogen, Carsbad, CA, USA) and amplified using primer pairs described in Table 1. Sequence data were assembled and edited with Seq Scape software v2.5 (Applied Biosystems). To determine the phylogenetic relationships, the sequences of the F gene were compared to the corresponding region of representative viruses of class II available in GenBank. Alignment and comparison of the nucleotide and amino acid sequences were performed using Clustal W in MEGA 5.0. Maximum likelihood (ML) trees were estimated using best-fit general time-reversible(GTR) model of nucleotide substitution with gamma-distributed rate variation among sites, and a heuristic SPR branch-swapping search available in PhyML version 3.0 (Guindon and Gascuel 2003). A bootstrap resampling process (100 replications) was employed to assess the robustness of individual nodes of phylogeny.

**Table 1:** **Primers set for amplification of complete F-gene**

Primers	Sequence 5’ to 3’
MF3710-F	TGA AAA CGA CGG CCA GTC AAA GCT GTA DGG TTG TG
MF4650-R	CAG GAA ACA GTA TGA CCA AGA GGC CTG CCR TCA A
F5100-F	TGA AAA CGA CGG CCA GTA TGC AGC ART TTG TYA AT
HF011-R	CAG GAA ACA GTA TGA CCT ARG TAA TRA GAG CRG ATG
HF005-F	TGA AAA CGA CGG CCA GTA GAC YGA AGG CGC ACT YAC
HH008-R	CAG GAA ACA GTA TGA CCA GRG CCA CYT GCT TRT ATA
F-4514-F	TGA AAA CGA CGG CCA GTG TAG AAG ADT YTG GAT CC
F-5218-R	CAG GAA ACA GTA TGA CCG AAT ACY GTA GTC AAY TCR G
HF009-R	CAG GAA ACA GTA TGA CCA GGT GGC ACG CAT ATT ATT
F-5757-F	TGA AAA CGA CGG CCA GTA GAT RAC AAC ATG TAG RTG
F-6449-R	CAG GAA ACA GTA TGA CCG GCT AAC YGC RCG GTC CAT

## Results

### Marketing practices

Chickens present at each market were estimated to be 300–500 on the days of visit. They are originated from different districts across the country, most of which were collected by traders from big towns such as: Sodo, Dessie, Shashemane, Jimma, Ambo, Hosaina and transported on public transport (bus) to Addis Ababa. The majority (99%) of traders mentioned that there is no habit of cleaning buses before loading and after unloading chickens owing to the fact that no vehicles is designed for poultry transportation alone. After arrival at the market sites, chickens are sold to wholesalers in the biggest live bird market site (Merkato) where birds from different origins are placed in the same cages (mixed) and sold either to traders in other live poultry markets in the city or to the consumers on spot. Ninety nine percent of the traders clean the holding cages once a week with clean water and the remaining 1% clean every day. All the traders responded that they dispose manure and dead birds either within the markets or on roadside. None of them seek for veterinary services when chickens are sick.

### Laboratory results

**Figure 2:**
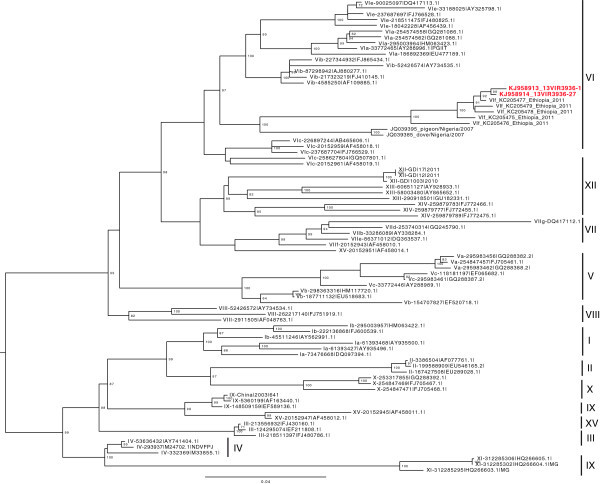
**Phylogenetic tree of the complete nucleotide sequence of the F gene for two representative isolates.** ML Phylogenetic tree of complete nucleotide sequence of Class II Newcastle diseases viruses. Ethiopian isolates included in this analysis (KJ958913_13VIR3936-1 and KJ958914_13VIR3936-27) are labeled in red. The Nomenclature system used in the phylogenetic tree is based on (Diel et al.2012). The numbers at branch points represent bootstrap values.

Out of the 146 pooled samples tested, 44 were positive for NDV by both HA/HI and M gene real-time RT-PCR tests. Out of these, 29 isolates were identified as virulent based on the real-time RT-PCR of F gene. Among these, 14 Tracheal swab pools (TS) were positive though the corresponding Cloacal swab pools (CS) were negative. Similarly, 3 CS were positive though the corresponding TS were negative. However, the remaining 12 swab pools have shown direct correlation (12 TS and the corresponding 12 CS were positive) and were not considered as separate entity to avoid double counting of the samples (Table 2). The identification, pathotyping and genotyping of the 29 isolates were confirmed by genetic sequencing of the 260 bp region of fusion (F) gene and the complete F gene sequence of two isolates (KJ958913_13VIR3936-1 and KJ958914_13VIR3936-27) collected from the market sites Saris and Kotebe. Since all the viruses showed a high similarity at the 260 bp region of the F gene (Table 2), the selection was based on the sequencing quality (i.e. absence of degenerate bases).The isolates were identified as virulent as deduced amino acid sequence of the fusion protein cleavage site indicated the presence of motif ^112^ RRQKR^116^*F^117^ typical of NDV virulent strains. Phylogenetic analysis of the 260 fragment of the F gene of all the 29 sequenced isolates (Additional file 1: Figure S1) and complete coding region of the F gene of two representative viruses (Figure 2) indicated that the viruses are clustered together (similarity ranged from 98% to 100%) within the new sub-genotype VIf.

**Table 2:** **Origin of positive swab pools and their genetic identity with reference sequence**

Market sites from where the samples were collected	Number of M-gene real time RT PCR positive swab pools	Number of F-gene(virulent) real time RT PCR positive swab pools				Genetic identity with the reference sequence at the 260 bp region of the F gene
		Total	Both TS &CS	Only TS	Only CS	
Shola	12	8	4	4	0	98 to 100%
Meri	9	5	4	1	0	98.7 to 100%
Saris	7	3	2	0	1	99.3 to 100%
Merkato	10	9	2	5	2	100%
Kotebe	6	4	0	4	0	100%

## Discussion

In this study, we assessed biosecurity practices in market sites in Addis Ababa that contribute to the spread of the diseases along market chain. We observed several practices that could promote Newcastle disease and other infectious diseases transmission among birds. These included limited cleaning and disinfection of the market places, holding cages, mixing of birds from different origins, inappropriate disposal of sick and dead birds and poor waste disposal mechanisms. Earlier reports indicated that marketing systems play a considerable role in the dissemination of diseases over wide geographical areas in a relatively short period of time in Ethiopia (Gebreab 1995). Similar observation was reported from Addis Ababa live poultry market sites (Shewantasew et al. 2012). The current finding is also in agreement with a report from Nairobi live bird markets where poor biosecurity practices were suggested to play major roles for transmission of avian influenza viruses (Munyua et al. 2012). These factors were found to be associated with transmission of low pathogenic avain influenza viruses in markets in North America (Garber et al. 2007).

The current study showed that, virulent viruses with multiple basic amino acids at cleavage site of F protein ^112^RRQKR^116^*F^117^ are circulating in village chickens. The finding is in agreement with earlier studies which revealed that, the majority of the virus strains circulating in the village chickens in Ethiopia are virulent strains (Chaka et al. 2012). Virulent Newcastle disease virus (vNDV) is endemic in many countries of Africa, North, Central, and South America, and outbreaks of ND are frequently reported to the World Organization of Animal health (Office of International Epizootes 2011).

According to Diel et al. (2012) classification, our phylogenetic analysis of the 260 fragment of the fusion gene of all the 29 sequenced isolates (Additional file 1: Figure S1) indicated that the viruses are grouped in the new sub-genotype VIf class II viruses, and grouped with NDV identified in Ethiopia (de Almeida et al. 2013; Chaka et al. 2013). The genotype was confirmed by analysis of complete coding region of the F gene of two representative viruses (Figure 2), which showed 95% to 99.3% similarity with the NDV isolated in 2011 suggesting the persistence of this sub-genotype in poultry in the country. While the 260 nucleotide sequences of samples collected from the market sites of Merkato and Kotebe were all identical, sequences of viruses from Saris, Shola and Meri showed a nucleotide identity ranging from 99.3% to 100%, from 98% to 100% and from 98.7% to 100%, respectively). Which revealed that, identical isolates are circulating in livebird markets in Addis Ababa. This could be associated with the poor biosecurity practices and the use of similar marketing channel in these market sites. However, Fentie et al. (2013) identified genotype VII viruses in the North western Ethiopia, indicating the co-circulation of different genotypes in Ethiopia. Genotype VI, include viruses that have been isolated from multiple avian species (Alexander 2011; Czeglédi et al. 2006; Kim et al. 2008). Viruses of this group are important because of their frequent association with doves and pigeons and consequent risk for introduction in to poultry flocks (Alexander 2011; Kim et al. 2008). It is interesting to note that Ethiopian viruses analyzed here grouped with viruses collected in Nigeria from pigeon and doves (identity of about 90%), suggesting that this genotype may have emerged in Africa as a result of transmission from pigeon to poultry.

In conclusion, the current study revealed that village chicken flocks are endemically infected with virulent Newcastle disease virus, which could pose a threat to commercial poultry farms. This study highlights the importance of implementing surveillance and molecular investigations of Newcastle disease, in village chickens and wild birds, along with improved biosecurity measures in the live poultry markets in Ethiopia.

## Electronic supplementary material

Additional file 1: **Phylogenetic tree of the 260 fragment of the F gene of all the 29 sequenced isolates.** (PDF 1 MB)
