# Supplementary figures and images for: Characterization of Newcastle Disease Virus and poultry-handling practices in live poultry markets, Ethiopia
Source: Springerplus. 2014 Aug 23;3:459. doi: 10.1186/2193-1801-3-459 (PMC4162888; doi:10.1186/2193-1801-3-459)

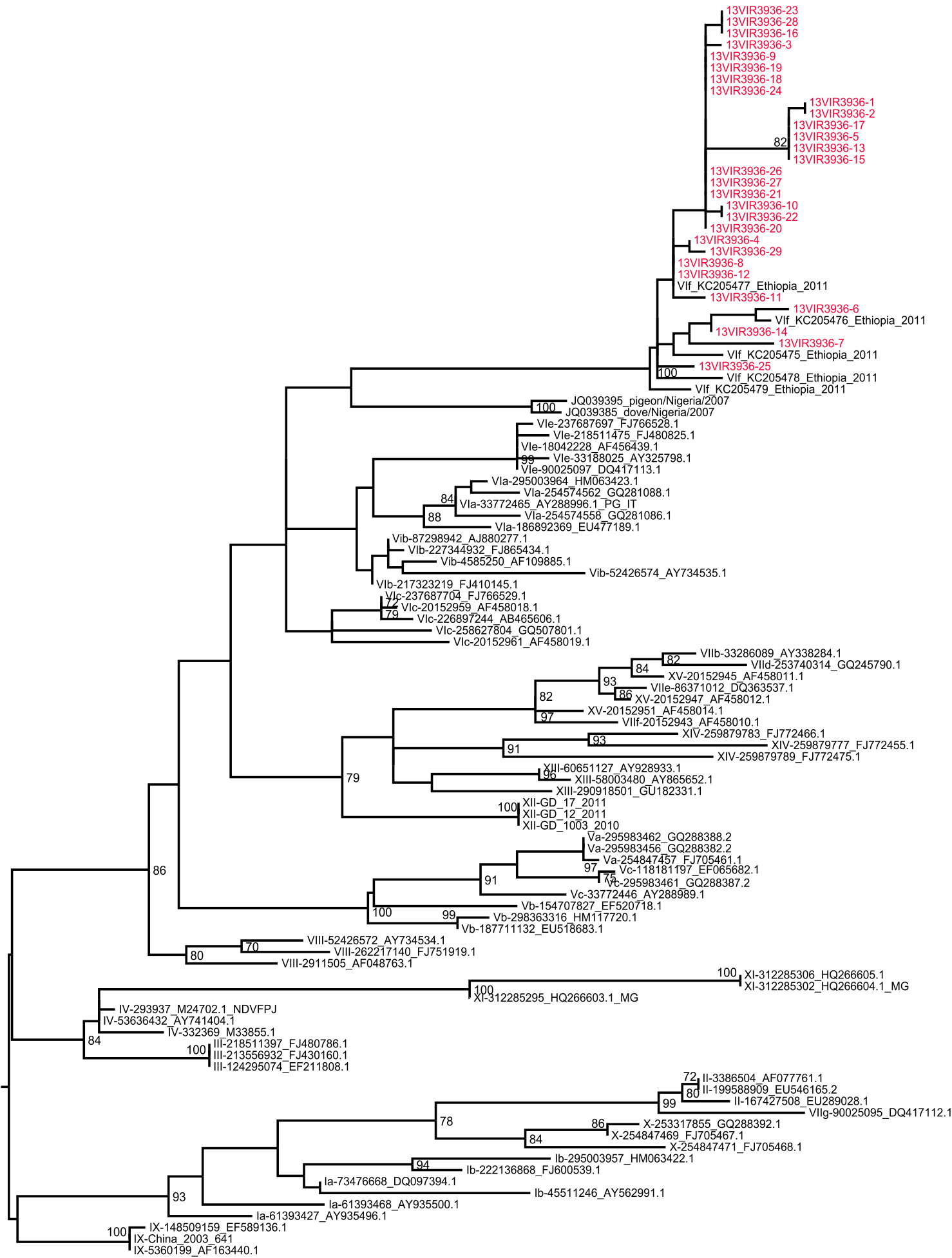

0.03

Supplement: Supplementary file 1 — Additional file 1: Phylogenetic tree of the 260 fragment of the F gene of all the 29 sequenced isolates. (PDF 1 MB) [file 40064_2014_1196_MOESM1_ESM.pdf]
